# Non-productive binding of cellobiohydrolase i investigated by surface plasmon resonance spectroscopy

**DOI:** 10.1007/s10570-021-04002-6

**Published:** 2021-08-25

**Authors:** Florian Csarman, Claudia Gusenbauer, Lena Wohlschlager, Gijs van Erven, Mirjam A. Kabel, Johannes Konnerth, Antje Potthast, Roland Ludwig

**Affiliations:** 1 Department of Food Science and Technology, Biocatalysis and Biosensing Laboratory, BOKU University of Natural Resources and Life Sciences, Muthgasse 18, 1190 Vienna, Austria; 2 Department of Materials Sciences and Process Engineering, Institute of Wood Technology and Renewable Materials, BOKU - University of Natural Resources and Life Sciences, Konrad-Lorenz-Straße 24, 3430 Tulln, Austria; 3 Laboratory of Food Chemistry, Wageningen University and Research, Bornse Weilanden 9, 6708 WG Wageningen, The Netherlands; 4 Department of Chemistry, Division of Chemistry of Renewable Resources, BOKU - University of Natural Resources and Life Sciences, Konrad-Lorenz-Straße 24, 3430 Tulln, Austria

**Keywords:** Cellobiohydrolase, Non-productive binding, Surface plasmon resonance, Biomass hydrolysis, Lignin, Glycosylation

## Abstract

Future biorefineries are facing the challenge to separate and depolymerize biopolymers into their building blocks for the production of biofuels and basic molecules as chemical stock. Fungi have evolved lignocellulolytic enzymes to perform this task specifically and efficiently, but a detailed understanding of their heterogeneous reactions is a prerequisite for the optimization of large-scale enzymatic biomass degradation. Here, we investigate the binding of cellulolytic enzymes onto biopolymers by surface plasmon resonance (SPR) spectroscopy for the fast and precise characterization of enzyme adsorption processes. Using different sensor architectures, SPR probes modified with regenerated cellulose as well as with lignin films were prepared by spin-coating techniques. The modified SPR probes were analyzed by atomic force microscopy and static contact angle measurements to determine physical and surface molecular properties. SPR spectroscopy was used to study the activity and affinity of *Trichoderma reesei* cellobiohydrolase I (CBHI) glycoforms on the modified SPR probes. N-glycan removal led to no significant change in activity or cellulose binding, while a slightly higher tendency for non-productive binding to SPR probes modified with different lignin fractions was observed. The results suggest that the main role of the N-glycosylation in CBHI is not to prevent non-productive binding to lignin, but probably to increase its stability against proteolytic degradation. The work also demonstrates the suitability of SPR-based techniques for the characterization of the binding of lignocellulolytic enzymes to biomass-derived polymers.

**Graphic abstract:**

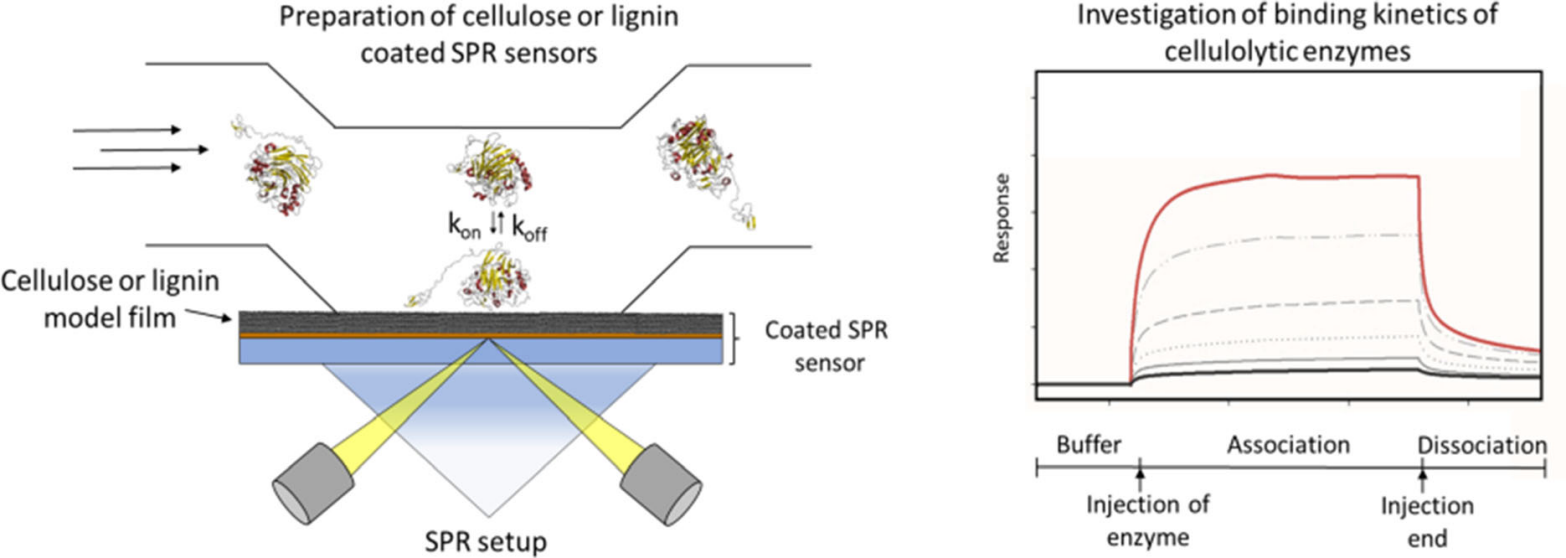

**Supplementary Information:**

The online version contains supplementary material available at 10.1007/s10570-021-04002-6.

## Introduction

The global climate crisis together with an increasing demand for energy and goods is the driving force of intensified research on sustainable resources. The efficient utilization of lignocellulosic biomass is considered to be a key technology for the transition from fossil resources towards a sustainable economy (Ragauskas et al. 2006; Chandel et al. 2018; De Bhowmick et al. 2018). The concept of modern biorefineries requests that the biopolymers found in lignocellulose are depolymerized and fractionated into fermentable sugars and chemical base stock. The bottleneck of the process is the recalcitrance of the plant cell wall against physical and chemical treatments (Ragauskas et al. 2014; Rinaldi et al. 2016). In comparison to acid- or alkali-based processes, enzymatic hydrolysis of lignocellulose is performed under mild reaction conditions (pH and temperature) that saves energy, avoids degradation of reaction products, and reduces the formation of undesirable side-products acting as inhibitors in subsequent fermentation processes (Dos Santos et al. 2016). Enzymatic hydrolysis at industrial scale is facing a variety of challenges including the limited efficiency of the enzymatic process, the variability in raw material composition requiring a different balance of enzymes, processing at high (solid) substrate loading, a long reaction time, and thereof resulting unfavourable process costs. A comprehensive understanding of the structure of plant biomass as well as of the molecular mechanisms, equilibrium constants and kinetic rates of the enzyme–substrate interaction is crucial to optimize enzymatic lignocellulose hydrolysis (Himmel et al. 2007; Merino and Cherry 2007; Dos Santos et al. 2016).

An interwoven network of the biopolymers cellulose, hemicellulose and lignin forms the basis of a plant cell wall. While cellulose is built from glucose subunits linked to a linear polymer via β-(1,4)-glycosidic bonds with a degree of polymerisation of up to 10 000 units, hemicelluloses represent a family of differently branched carbohydrate polymers composed of hexoses and pentoses with a degree of polymerisation of 50–300 residues (Pu et al. 2008; Teleman 2009; Heinze 2015). In contrast to the completely amorphous hemicelluloses, cellulose chains are able to interact to regular semi-crystalline fibres and fibrils stabilized by hydrogen bond networks and van der Waals forces.

Lignin is an irregular polymer formed by the free radical polymerisation of the aromatic monolignol precursors coniferyl, sinapyl and p-coumaryl alcohols giving rise to a network composed of C–C or C–O-linked guaiacyl- (G), syringyl- (S), or *p*-hydroxyphenyl (H) subunits, respectively. The ratio of these subunits differs between individual plant species. While lignin found in herbaceous biomass consists of G, S and H subunits and includes hydroxycinnamic acids, hardwood lignin consists of only G and S units while softwood lignin is almost exclusively composed of G units with small amounts of H subunits (Schutyser et al. 2018; Lourenço and Pereira 2018). Many studies have been devoted to understanding the relation between these fundamentally different lignin structures and overall cell wall recalcitrance towards chemical and enzymatic degradation. However, the underlying mechanisms are still a matter of debate (Huntley et al. 2003; Yoo et al. 2018; Zoghlami and Paës 2019).

Lignin forms a major barrier that limits accessibility of cellulose and hemicellulose by hydrolytic enzymes and additionally, lignin can bind cellulases in an unspecific manner reducing the amount of available active enzymes. (Jeoh et al. 2007; Álvarez et al. 2016; Siqueira et al. 2017) This non-productive binding reduces the efficiency of enzymatic processes. A variety of pretreatment procedures intend to reduce non-productive binding of enzymes by selective lignin removal (Ding et al. 2012) or by thermochemical modification of lignin (Sipponen et al. 2017) to decrease enzyme association with lignin. The development of cellulase preparations less affected by lignin and reduced non-productive binding is suggested as an alternative approach to improve hydrolytic enzyme cocktails (Berlin et al. 2005, 2006).

For lignocellulose saccharification a variety of processive cellobiohydrolases (CBHs), endo-β-4-glucanases (EGs), and β-glucosidases supported by hemicellulose-active hydrolases and esterases as well as auxiliary oxidoreductases like lytic polysaccharide monooxygenase (LPMOs) are used to synergistically convert carbohydrate polymers to monosaccharides (Rosgaard et al. 2007; Vaaje-Kolstad et al. 2010; Horn et al. 2012). Although there is some evidence by MD simulations and experimental work that non-productive binding of enzymes to lignin is governed primarily by hydrophobic interactions (Palonen et al. 2004; Chen et al. 2011; Pareek et al. 2013; Rahikainen et al. 2013; Sammond et al. 2014) some other studies also indicate the importance of hydrogen bonding (Pan 2008) and electrostatic interactions (Nakagame et al. 2011) between lignin and biomass degrading enzymes. A common posttranslational modification to increase solubility and decrease the hydrophobic surface properties of extracellular proteins is N- and O-glycosylation. This protein modification found across eukaryotic species is also involved in efficient secretion, thermal stability, catalytic activity, stability against proteolytic degradation and increase the solubility and hydrophilicity. However, the implications of glycosylation on the binding behaviour of lignocellulolytic enzymes and especially on the interaction with lignin are still not completely clear and experimental challenges interfere with the studies.

Surface plasmon resonance (SPR) spectroscopy represents a surface-sensitive method allowing for the label-free quantification and characterisation of bimolecular interactions in real-time. Today SPR-based methods are commonly applied in different fields including drug development and screening for pharmaceutically active compounds (Patching 2014; Olaru et al. 2015), antibody engineering (Alfthan 1998; DiCara et al. 2018), or clinical analytics and food safety (Piliarik et al. 2009; Situ et al. 2010; Li et al. 2012; Mariani and Minunni 2014). SPR biosensors modified with cellulose thin films have been used to study adsorption behaviour dependent on pH, temperature and ionic strength. Furthermore, the importance of the carbohydrate binding module (CBM) of endoglucanase GH45 from *Humicola insolens* for substrate binding and efficient catalytic turnover was demonstrated by using cellulose model films on SPR probes (Eriksson et al. 2005a, 2005b). Lignin-modified SPR probes have been successfully employed for the study of the binding and precipitation behaviour of glycinin and β-conglycinin from soy beans (Salas et al. 2013). An SPR probe modified with milled wood lignin immobilized onto a self-assembled monolayer of methoxypolyethylene glycol thiol (mPEG-SH) was used to investigate the binding of 12-mer peptides to lignin (Yamaguchi et al. 2016; Isozaki et al. 2018)**.**

In this study, we characterize the binding behaviour of cellulolytic enzymes, especially CBHI, to lignin and cellulose films on SPR probes used as model systems. This experimental setup was applied to investigate the role of N-glycosylation of CBHI from *Trichoderma reesei*, an important posttranslational modification of extracellular enzymes, for specific binding to cellulose and its proposed function to reduce non-productive binding of enzymes to the types of lignin used.

## Materials and methods

### Materials and chemicals

Unless stated otherwise all chemicals and reagents were purchased from Sigma-Aldrich (St. Louis, MO, USA) or Carl Roth (Karlsruhe, GER) and were of highest purity available. High quality water (HQ-water, > 18 MΩ cm^−1^) for the preparation of solutions and buffers was prepared using a SG System (Barsbüttel, GER). Microcrystalline cellulose (MCC) for column chromatography was purchased from Merck (Darmstadt, GER) and particles were fractionated by size using a stack of analytical sieves (Haver&Boecker, Oelde, GER). Phosphoric acid swollen cellulose (PASC) was prepared from MCC with a particle size of 45–125 µm and following a published procedure with modifications (Wood 1988; Kracher et al. 2018). In brief, 4 g of MCC were treated with 100 mL ice-cold phosphoric acid (85% w/w) and stirred at 4 °C for 18 h. The reaction mix was filled to 2.0 L with cold HQ-water to precipitate cellulose. The precipitate was recovered by vacuum filtration and washed with 2.0 L of cold HQ-water, 2.0 L of sodium bicarbonate (2.0 M) and 1.0 L sodium phosphate buffer (50 mM, pH 6.0). Finally, PASC was diluted with HQ-water and further treated with an Ultra Turrax (Ika, Staufen, GER) to obtain a homogenous solution. Hardwood organosolv lignin (OSL) was prepared from beech (*Fagus sp.*) wood with a mass average molar mass of M_w_ of 10 000 g mol^−1^ and a dispersity (Ð, M_w_/M_n_) of 3.1 as reported previously (Zinovyev et al. 2018). Milled Wood Lignin (MWL) from spruce (*Picea abies*) was prepared as described previously by Björkman with modifications (Björkman 1954; Van Erven et al. 2019). In brief, removal of extractives was achieved by sequential extraction with acetone and water, the resulting-extractive free sample was finely milled in a planetary ball-mill. After water extraction and freeze-drying, extraction of lignin was performed in aqueous dioxane (80% v/v) for 24 h at room temperature under nitrogen atmosphere twice. Non-soluble material was subsequently separated by centrifugation and the solvent was removed from the lignin sample by freeze-drying.

### Enzyme preparation

Celluclast® 1.5L from Novozymes (Cellulase from *Trichoderma reesei* ATCC 26921) purchased via Sigma Aldrich was used as a commercial enzyme mixture and for the purification of CBHI. The purification procedure was adapted from the published protocol using two consecutive steps of column chromatography (Jäger et al. 2010). For the first step of purification by anion exchange chromatography, 10 mL Celluclast were rebuffered to 50 mM Tris–HCl, pH 7.0 using 60 mL of Sephadex G-25 Fine (GE Healthcare, Chicago, IL, USA) and the sample was loaded on a 30 mL DEAE-Sepharose column (XK16, GE Healthcare) equilibrated with the same buffer. Elution was performed stepwise using 35% and 100% elution buffer (200 mM NaCl in 50 mM Tris–HCl, pH 7.0). CBHI eluting at 100% elution buffer was concentrated using a VivaFlow 50 module with a PES membrane and a molecular weight cut-off (MWCO) of 10 kDa (Sartorius, Goettingen, GER) and was finally purified by size exclusion chromatography using Sephacryl S200 (XK16, 60 cm, GE Healthcare) equilibrated with 10 mM sodium acetate buffer, pH 4.8 applying 0.5 mL of the concentrated protein solution at a flowrate of 0.5 mL min^−1^. Glyoxal oxidase from *Phanerochaete chrysosporium* (Wohlschlager et al. 2021b), cellobiose dehydrogenase from *P. chrysosporium* (Wohlschlager et al. 2021a) and laccase from *Botrytis aclada* (Kittl et al. 2012) were produced and purified as described previously.

Deglycosylation was achieved using 1000 U of Endo Hf (New England Biolabs, MA, USA) per mg of enzyme and incubation at 30 °C for 18 h in 50 mM sodium acetate buffer, pH 6.0. Endo Hf was removed using an MBPTrap HP column (GE Healthcare) equilibrated with binding buffer containing 20 mM Tris–HCl buffer, pH 7.4 containing 200 mM NaCl and 1 mM EDTA,. The flow-through containing the deglycosylated proteins was collected and bound Endo Hf was afterwards eluted in the same buffer supplemented with 10 mM maltose.

All enzyme preparations were rebuffered to 20 mM sodium acetate buffer, pH 5.0 supplemented with 0.05% Tween 20 using PD 10 desalting columns (GE Healthcare). The purity of the enzyme preparations was evaluated by SDS-PAGE using a 4–20% Mini-PROTEAN® TGX stain-free™ precast gel (BioRad Hercules, CA, USA) and Precision Plus Protein Unstained Standard (BioRad) as ladder for the determination of the molecular weight. Results were evaluated using a Gel Doc XRS + system and Image Lab 6.1 (BioRad) for analysis.

### Atomic force microscopy (AFM)

AFM images of cellulose- and lignin modified surfaces as well as of different intermediate steps of the modification procedure were obtained using a Dimension Icon Scanning Probe Microscope (Nanoscope V, Bruker, Santa Barbara, CA, USA) equipped with a silicon tip on a silicon nitride cantilever (ScanAsyst-Air, Bruker) with a resonance frequency of 70 kHz and a nominal spring constant of 0.4 N m^−1^. Spring constant and deflection sensitivity were calibrated prior imaging in the Bruker software. Topography and Peak Force Error images were obtained in Peak Force Quantitative Nanomechanical Mapping Mode (Peak Force QNM®, Bruker) with a scan speed in the range between 0.153 and 0.312 Hz collecting 256 or 512 pixels per line. Image processing as well as the evaluation of height profiles and surface roughness was performed using Gwyddion 2.56 (Nečas and Klapetek 2012). The raw data was treated with mean plane subtraction and row alignment before the surface roughness was evaluated from at least three image areas (5 × 5 µm).

### Determination of static contact angles and surface free energy (SFE)

Static contact angles were measured at 23 °C and 55% humidity using a Drop Shape Analyzer DSA 30S (Krüss GmbH, Hamburg, GER). Purified water, ethylene glycol and diiodomethane were used as test liquids. Droplets of 1.0 µL were deposited on the surface and images were collected at 10 fps for 2 s. Contact angles were determined using the sessile drop method by fitting the drop shape as ellipse and setting the baseline automatically by the built in software Advance (Krüss). Surface free energy was calculated according to the Owens–Wendt-Rabel-Kaelble (OWRK) method (Owens and Wendt 1969; Kaelble 1970).

### Determination of enzymatic activity

The hydrolytic activity was determined measuring the release of reducing sugars based on the method by Nelson and Somogyi (Nelson 1944; Somogyi 1952). MCC (10 g L^−1^), PASC (5 g L^−1^) carboxymethylcellulose (CMC, 10 g L^−1^) and α-cellulose (10 g L^−1^) were used as substrates. MCC and PASC were prepared as described previously, CMC sodium salt with a degree of substitution of 0.60–0.95 was purchased from FLUKA and α-cellulose from Sigma-Aldrich. All reactions were performed in 20 mM sodium acetate buffer, pH 5.0. The substrate was incubated with the enzyme solution in a final volume of 250 µL in 2 mL tubes for 60 min at 30 °C. Blanks containing only the substrate were prepared equivalently. The reaction was stopped by boiling for 5 min and the remaining substrate was precipitated by centrifugation at 20238 × g for 3 min. The clear supernatant (200 µL) was mixed with 200 µL Somogyi solution and incubated for 20 min at 99 °C in the water bath. After cooling, 200 µL Nelson solution and 2.3 mL HQ-water were added. The absorbance at 540 nm was determined using an Agilent 8453 UV–visible spectrophotometer (Agilent Technologies, Santa Clara, CA, USA) and the concentration of released reducing sugars was calculated using a calibration curve obtained with glucose. One unit was defined as one µmol glucose equivalents produced per min under the assay conditions.

### Binding studies with MCC and PASC

Binding of CBHI and Celluclast 1.5L to MCC and PASC was determined in 20 mM sodium acetate buffer, pH 5.0 at 30 °C. Different concentrations of CBHI (1–50 µM) were incubated with 10 mg mL^−1^ MCC (particle size < 45 µm) or 1.25 mg mL^−1^ PASC in a thermomixer at 800 rpm for 30 min and the enzyme bound to the substrate was removed by centrifugation at 20238 × g for 3 min. For purified CBHI the concentration of remaining enzyme in the supernatant was determined spectrophotometrically measuring the absorbance at 280 nm using an Agilent 8453 UV–visible spectrophotometer. The dissociation constant (*K*_*D*_) and maximum binding capacity (*B*_*max*_) was calculated by fitting the data to a hyperbolic function using Sigma Plot 14.0 (Systat Software Inc., San Jose, CA, USA).

### Preparation of cellulose and lignin films

The procedure for the dissolution of cellulose in N,N-dimethylacetamide (DMAc) and the preparation of homogenous cellulose films was adapted from previously published procedures (Raj et al. 2012; Sczech and Riegler 2006). In brief, MCC was swollen in HQ-water constantly stirred at 22 °C for 18 h and further incubated in methanol for 45 min at 40 °C twice. The material was treated with DMAc at 22 °C for 45 min for four times. Activated cellulose was finally dried at 60 °C under vacuum for 24 h and dissolution was performed using anhydrous DMAc containing 7% (w/w) water free LiCl under constant stirring at 40 °C for 18 h.

Cellulose films on SPR probes were produced as previously described using the SIA Kit Au (GE Healthcare) (Laurent et al. 2019). The gold surface was cleaned with alkaline Piranha solution (NH_4_OH/H_2_O_2_/H_2_O, 1:1:3) at 75 °C for 15 min, washed with HQ-water and dried at 80 °C. The surface was then pretreated with 100 mg L^−1^ poly(diallyldimethylammoniumchloride) (PDADMAC, Sigma Aldrich) as adhesion promoter supplemented with 10 mM NaCl for 30 min. After washing in HQ-water and drying, activated cellulose was deposited by spin coating using 80 µL of 0.5 (w/w) cellulose in DMAc/LiCl at 3000 rpm for 3 min. The solvent was removed by drying at 160 °C and the resulting cellulose film was washed extensively in HQ-water to remove LiCl. Finally, the modified gold surface was dried at 160 °C and stored at room temperature in the desiccator over silica.

The preparation of lignin films using OSL from beech wood or MWL from spruce was adapted from a published procedure (Pereira et al. 2017). The lignin preparations were dissolved to a final concentration of 0.5% (w/w) in 1,4-dioxane/water (96:4, v/v) under rotary shaking at 22 °C for 24 h. The gold surface was cleaned as described previously and modified with 0.5% (w/v) polystyrene (PS, average Mw 35 000, Sigma Aldrich) dissolved in toluene by spin coating at 2000 rpm for 30 s. Lignin was deposited 8 times using the same conditions for spin coating and the layer was dried at 80 °C for 30 min between each step. The modified SPR probe was stored at room temperature in a desiccator over silica under nitrogen atmosphere until further use.

### SPR measurements

The SPR probe was assembled according to the instructions of the manufacturer. SPR measurements were performed using a Biacore T200 system (GE Healthcare) with 20 mM sodium acetate buffer, pH 5.0 supplemented with 0.05% (w/w) Tween 20 used as running buffer. Experiments were performed at 30 °C with a flow rate of 10 µL min^−1^ and for the regeneration of the sensor surface a 5 M sodium chloride solution for 60 s and a 10 mM glycine/HCl buffer, pH 2.0 for 30 s at the same flow rate was used. For the initial equilibration of the surface, three cycles of blank injections with running buffer followed by regeneration of the sensor surface was performed. The equilibration was performed to guarantee for a stable baseline during the measurements and to avoid effects caused by ongoing swelling of the sensor surface or other surface rearrangements. Binding kinetics using cellulose were determined by single-cycle-kinetic (SCK) measurements with five sequential injections of the analyte with an association period of 60 s and a final dissociation period of 180 s without regeneration between the injections of one cycle. Binding to lignin surfaces was investigated by multi-cycle-kinetic (MCK) measurements with an association period of 120 s and a dissociation period of 180 s. All samples were rebuffered using PD-10 desalting columns (Sephadex G-25 M, GE Healthcare) and diluted in running buffer to avoid differences in refractive index of the solvent. Data were evaluated using the BiaEvaluation Software version 3.1 (GE Healthcare) and SigmaPlot version 12.0.

The thickness (d) of the adsorbed film was estimated according the method by Jung et al. from the SPR response R evaluating the slope m obtained from a calibration curve using glycerol solutions with known refractive index (Fig. S5) and the difference between the refractive index of the adsorbed layer (η_a_) and the refractive index of the solvent (η_S_) (Eq. 1) (Jung et al. 1998; Pereira et al. 2017; Salas et al. 2013).

1 $$ {\text{d}} = \frac{{l_{d} }}{2}\frac{R}{{m~\cdot\left( {\eta _{a}  - ~\eta _{S} } \right)}} $$

The characteristic decay length of the evanescent wave (l_d_) was approximated as 0.37 times the light wavelength, the refractive indices for cellulose, lignin, the adsorbed protein layer, and the bulk fluid were estimated as 1.47, 1.61, 1.44, and 1.333, respectively (Jung et al. 1998; Vörös 2004; Norgren et al. 2006; Kasarova et al. 2007) (Fig. S5).

The surface loading (Γ) was calculated using the density (ρ) of the adsorbed layer with ρ of cellulose assumed as 1.60 g cm^−3^ and lignin as 1.30 g cm^−3^ (Eq. 2, Daicho et al. 2020; Triwulandari et al. 2019). For the calculation, the densities reported for dry and homogenous compounds were used. The density of the adsorbed protein was estimated as 1.32 g cm^−3^ according to Eq. 3 using the molecular weight (M), the volume of the protein (V) in Å^3^ and Avogadro’s number (N_A_) (Quillin and Matthews 2000).

2 $$ \Gamma  = {\text{d}}\rho $$

3 $$ \rho  = \frac{{10^{{24}} ~M~}}{{V~N_{A} }} $$

A model of the full-length CBHI obtained with SWISS-MODEL (https://swissmodel.expasy.org/) using the structures of the catalytic domain (pdb: 7CEL) and its CBM (pdb: 5×34) as template was used to calculate the molecular volume according to the Voronoi procedure applying VADAR Version 1.8 (Willard et al. 2003).

## Results and discussion

### Characterisation of cellulose and lignin films

Cellulose films from MCC dissolved in DMAc/LiCl and lignin films using MWL (spruce) or OSL (beech) dissolved in dioxane/water were prepared by spin-coating. MWL and OSL from softwood and hardwood respectively were chosen to represent lignin preparations with different characteristics. While MWL from spruce is almost exclusively composed of G-units and the extraction procedure prevents extensive alterations of the naturally occurring lignin, OSL from beech wood is composed of mainly S and G-units and is substantially modified and condensed during the extraction.

**Fig. 1:**
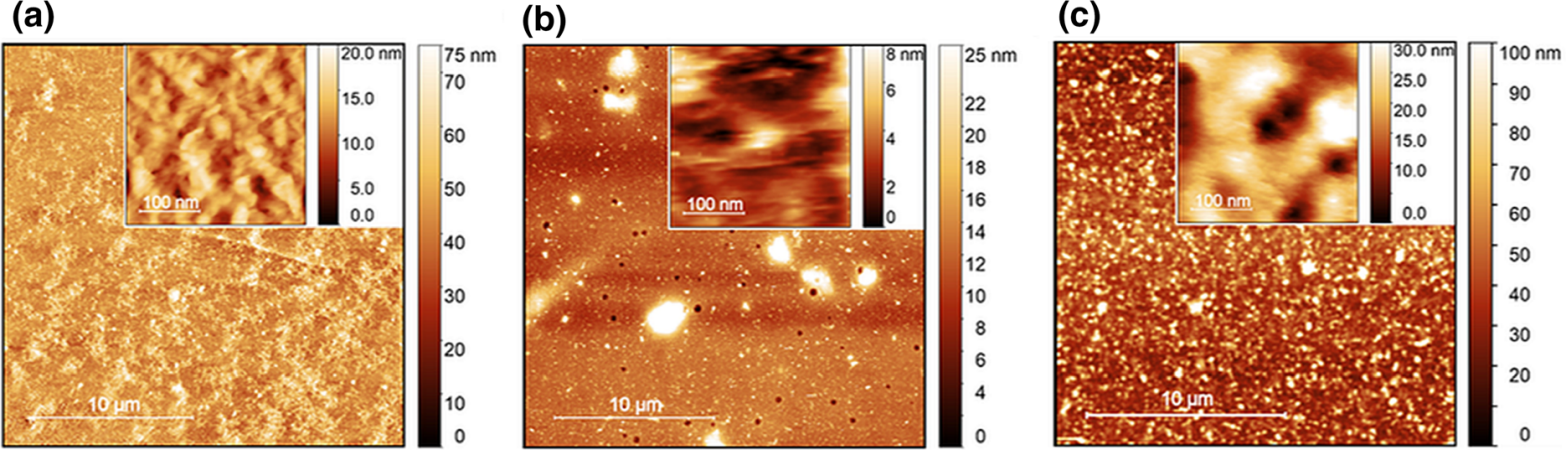
AFM images of dry cellulose (**a**), MWL (**b**) and OSL (**c**) films prepared on the gold surface of SPR probes. Scale bars represent 10 µm in main images and 100 nm in insets

Initial experiments were performed using glass slides to optimize the spin-coating time, angular velocity, and substrate concentration for the preparation of homogenous films. Based on the optimized parameters, films were prepared on SPR probes. Prior to the modification with the substrate, the surface was modified with polycationic PDADMAC for cellulose films or with polystyrene (PS) for lignin films to promote adhesion. The different steps of modification and the final films were analysed by AFM imaging (Fig. S1). All films on SPR probes were shown to be homogenous and to cover the entire measurement area (Fig. 1). The root mean square (RMS) roughness measured from at least three areas with a size of 25 µm^2^ was calculated and showed that the roughness of the cleaned glass or gold surface increased when modified with PDADMAC or PS (Table 1). Modification of surfaces with cellulose or lignin resulted in a further increase of roughness to 5.60 ± 1.54 nm for cellulose, 4.44 ± 0.01 nm for OSL and 8.30 ± 1.72 nm for MWL on the SPR probes. The RMS roughness of the cellulose surface is comparable to that reported for cellulose films regenerated from cellulose xanthate with 4.99 to 5.17 nm (Weißl et al. 2018) or films prepared from cellulose dissolved in DMAc with 4.8 nm (Table S1) (Eriksson et al. 2005a). Spherical aggregates were visible in the AFM images of the lignin films as observed in prior studies using MWL from different sources and Kraft lignin for the preparation of artificial lignin films. (Norgren et al. 2006; Notley and Norgren 2010; Pereira et al. 2017) Although the observed RMS roughness is slightly higher than observed in the cited studies, the distribution of nanoscale particles appeared uniform and the film covered the underlying surface homogenously.

**Table 1:** Static contact angles and surface roughness of modified and non-modified gold and glass surfaces. Surface roughness was calculated from AFM images using Gwyddeon 2.56. Data represent mean values and standard deviations of at least three areas (5 × 5 µm)

Surface	Modification	Static contact angle [deg]			Surface roughness [nm]	
		Water	Ethylenglycol	Diiodo-methane	RMS roughness (Sq)	Mean roughness (Sa)
Gold	None	89.9 ± 0.3	64.8 ± 0.4	60.4 ± 0.2	0.39 ± 0.02	0.31 ± 0.01
	PDADMAc	70.3 ± 4.9	49.1 ± 0.2	47.6 ± 0.2	1.68 ± 0.08	1.20 ± 0.07
	PS	103.5 ± 0.6	76.8 ± 1.6	18.7 ± 2.3	2.33 ± 0.62	0.99 ± 0.18
	OSL	67.6 ± 0.6	37.9 ± 3.7	34.2 ± 0.1	4.44 ± 0.01	3.26 ± 0.07
	MWL	68.3 ± 1.2	43.4 ± 1.7	41.6 ± 0.1	8.30 ± 1.72	2.80 ± 0.21
	Cellulose	30.5 ± 0.1	16.2 ± 20.9	45.1 ± 0.2	5.60 ± 1.54	3.91 ± 0.90
Glass	None	72.1 ± 0.8	63.1 ± 0.1	64.8 ± 0.3	0.80 ± 0.14	0.51 ± 0.07
	PDADMAc	58.3 ± 0.1	44.5 ± 0.4	48.3 ± 0.2	2.13 ± 0.39	1.03 ± 0.09
	PS	90.5 ± 0.1	68.3 ± 0.2	14.9 ± 3.4	1.29 ± 0.01	0.85 ± 0.01
	OSL	64.0 ± 0.9	40.3 ± 3.2	39.6 ± 0.1	n.d	n.d
	MWL	62.2 ± 1.0	38.8 ± 2.1	37.2 ± 0.2	n.d	n.d
	Cellulose	30.0 ± 0.4	23.2 ± 3.7	43.3 ± 0.1	5.42 ± 1.15	4.19 ± 0.90

To characterize the hydrophobicity and wettability of the films, contact angle measurements were performed using water, ethylene glycol, or diiodo-methane as testing liquids. SPR probes modified with cellulose showed a static contact angle of 30.5° ± 0.1° and 45.1° ± 0.2° with water and diiodo-methane, respectively, which is consistent with the values reported for cellulose films produced using DMAc/LiCl as solvent, regenerated from cellulose xanthate or trimethylsilyl cellulose (Eriksson et al. 2005a; Sczech and Riegler 2006; Mohan et al. 2011; Weißl et al. 2018). For cellulose-modified gold and glass surface, similar contact angles were observed. The water contact angles determined for OSL and MWL were 67.6° ± 0.6° and 68.3° ± 1.2°, respectively, and are in excellent agreement with the values (66–69°) reported for MWL from different sources for the preparation of thin films on gold (Pereira et al. 2017). For OSL prepared from beech wood a slightly lower water contact angle of 58° ± 1° was determined on PS-coated silicon wafers (Borrega et al. 2020). Thin films prepared from Kraft lignin were shown to result in even lower contact angles highlighting the importance of the performed lignin extraction process for the characteristics of the resulting film (Norgren et al. 2006).

**Fig. 2:**
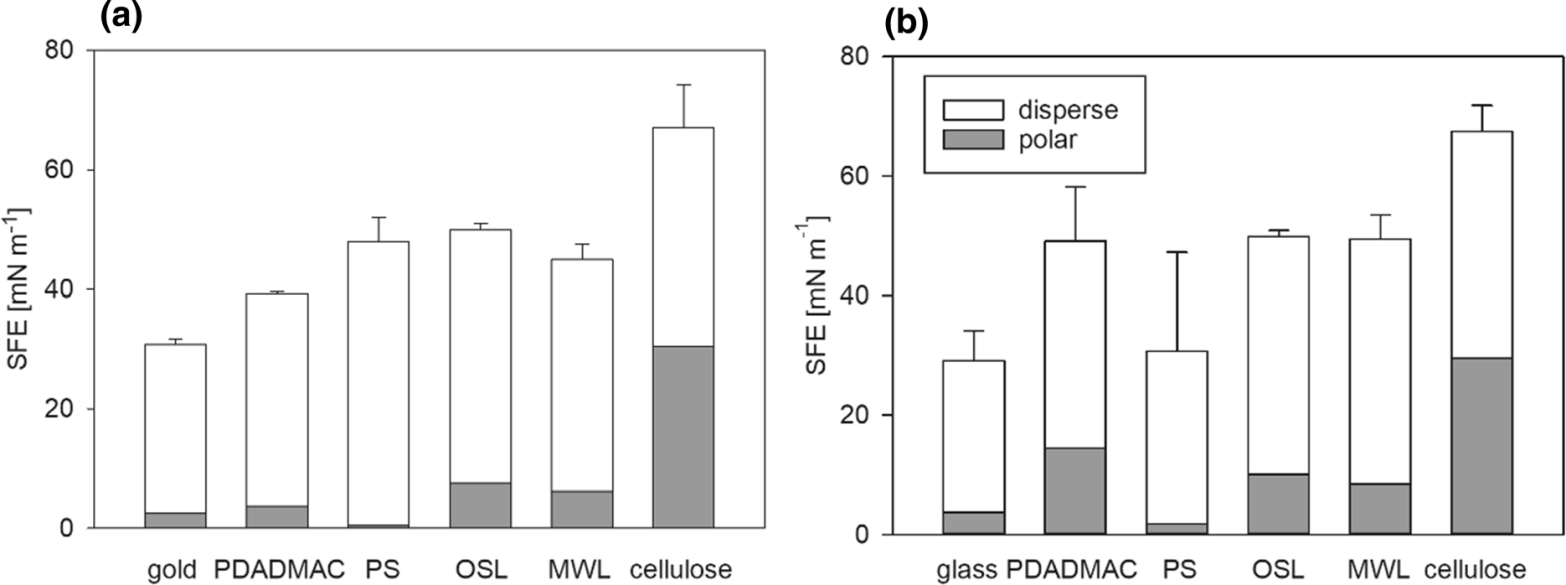
Sum of surface free energy from disperse and polar contributions for cellulose and lignin films prepared on gold (**a**) and glass (**b**) surfaces

The polar and dispersive components of the surface free energy (SFE) were calculated according to the Owens–Wendt-Rabel-Kaelble model (Fig. 2). The SFE of the gold or glass surfaces increases when treated with PDADMAC or PS and is further increased by the final modification with cellulose or lignin. While the total SFE of the lignin films is predominantly determined by the disperse component, for the cellulose film the polar component is almost equal. The values determined within this study are in the range of previous works and indicate a hydrophilic surface of the cellulose film (Table SI 1) (Eriksson et al. 2007; Notley and Norgren 2010; Weißl et al. 2018).

### Hydrolytic activity with different cellulosic model substrates

**Fig. 3:**
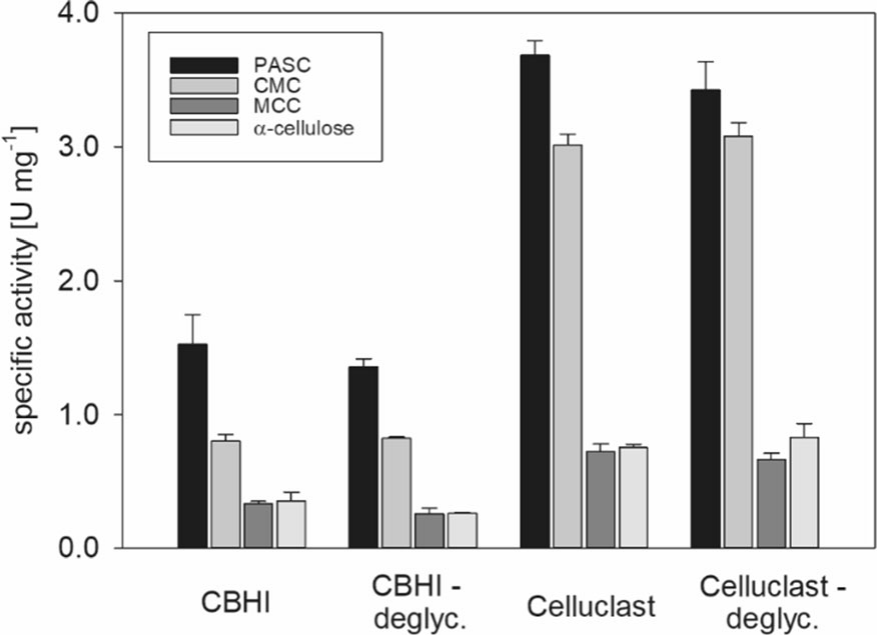
Comparison of cellulose hydrolysis in solution by CBHI and Celluclast before and after deglycosylation with Endo Hf using PASC, CMC, MCC and α-cellulose as substrates

Celluclast and purified CBHI were deglycosylated using Endo Hf and the enzyme used for deglycosylation was subsequently removed using an MBPTrap column. The homogeneity of the enzyme preparation and the successful removal of Endo Hf were demonstrated by SDS-PAGE. (Fig. S2) The activity of untreated and deglycosylated enzyme preparations were assayed by the release of reducing sugars from different model substrates and quantified by the method of Nelson and Somogyi using a calibration curve obtained with D-glucose (Fig. S3). With each enzyme preparation, the highest activity was found using PASC as substrate while the activity observed with MCC and α-cellulose was lower by a factor of 4.6 and 5.2, respectively (Fig. 3). This difference in hydrolytic activity shows the large influence of cellulose crystallinity on its efficient enzymatic degradation (Percival Zhang et al. 2006; Hall et al. 2010). The yield of reducing sugars using Celluclast exceeded that of purified CBHI by a factor of 2.1–2.6 with the model substrates PASC, MCC, and α-cellulose. For CMC a 3.7–3.8 higher activity could be observed using Celluclast compared to isolated CBHI. This high difference in specific activity can be explained by the good susceptibility of this substrate for degradation by EGs (Claeyssens and Aerts 1992). No significant difference in hydrolytic activity could be detected between the deglycosylated and untreated enzyme preparations. The results demonstrate that the N-glycosylation of CBHI is not influencing enzyme activity and that the enzymes maintain sufficient stability upon removal of N-linked glycans under the chosen reaction conditions in these experiments (30 °C, 1000 rpm, 60 min).

### Binding studies and adsorption isotherms

**Fig. 4:**
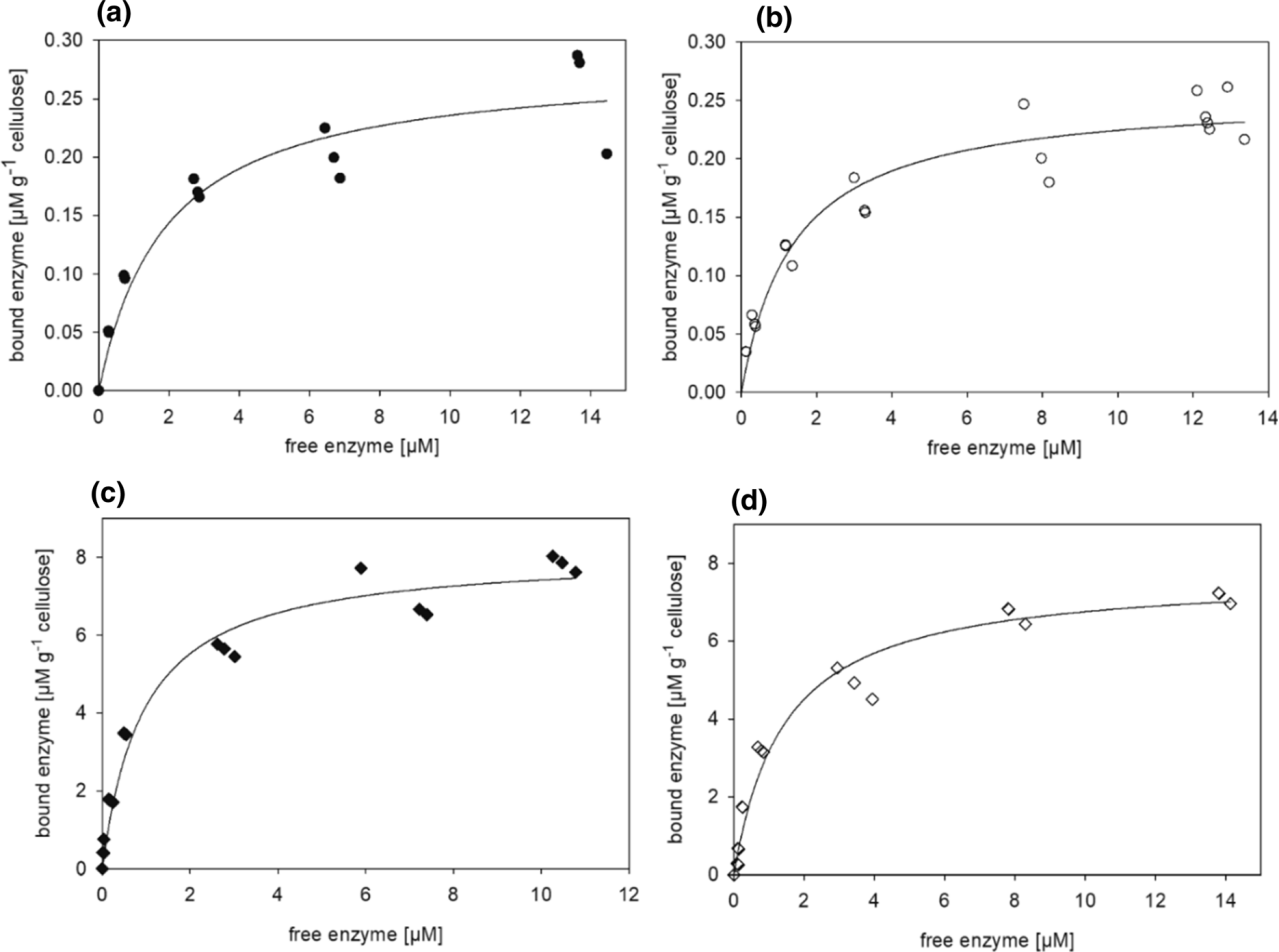
Binding of untreated CBHI to MCC (**a**) and PASC (**c**) compared to the Endo Hf treated, deglycosylated CBHI binding to MCC (**b**) and PASC (**d**). Data were fitted according to the Langmuir model to a hyperbolic equation using SigmaPlot 14.0

Substrate binding is a crucial, potentially rate limiting step of enzyme catalysis. Binding experiments of the untreated and deglycosylated CBHI in solution were performed with amorphous PASC and MCC to study the influence of N-glycans on substrate binding (Fig. 4). All reactions were incubated for 30 min to reach the binding equilibrium and to avoid excessive substrate degradation. Data were fitted according tothe Langmuir model to a hyperbolic function with *B*_max_ being the maximum binding capacity at equilibrium conditions, *K*_D_ representing the dissociation constant, and [E] the concentration of enzyme (Eq. 4).

4 $$ \left[ {\text{E}} \right]_{{adsorbed}}  = \frac{{B_{{{\text{max}}}} ~*~\left[ {\text{E}} \right]}}{{K_{{\text{D}}}  + \left[ {\text{E}} \right]}} $$

While the dissociation constant of CBHI for PASC slightly increased from 0.94 ± 0.17 to 1.43 ± 0.25 µM upon deglycosylation, no significant difference between the *K*_D_ and *B*_max_ was observed between the untreated and deglycosylated CBHI using MCC (Table 2). Henriksson et al. reported a slightly higher *K*_D_ of CBHI for MCC of 3.6 µM. However, those measurements were performed at a 5 °C lower temperature and in 50 mM ammonium acetate buffer, pH 5.0, compared to 20 mM sodium acetate buffer, pH 5.0, used in our study (Henriksson et al. 1997).

**Table 2:** Dissociation constants (*K*_D_) and maximum binding capacity (*B*_max_) of glycosylated and deglycosylated CBHI evaluated by using the Langmuir equation

Enzyme preparation	Substrate	*K*_D_ [µM]	*B*_max_ [µmol g^−1^cellulose]	R^2^
Untreated CBHI	PASC	0.94 ± 0.17	8.11 ± 0.36	0.96
	MCC	1.91 ± 0.58	0.28 ± 0.02	0.90
Deglycosylated CBHI	PASC	1.43 ± 0.25	7.72 ± 0.35	0.96
	MCC	1.40 ± 0.23	0.25 ± 0.01	0.96

The binding of CBHI to cellulose is a complex process influenced on the one hand by the heterogeneity of the substrate considering the differences between crystalline and amorphous regions as well as degree of polymerization and accessibility of chain ends, and on the other hand by the molecular architecture of the enzyme consisting of a catalytic domain and a CBM connected by a flexible linker. A variety of different binding models have been proposed including the Freundlich model, combined Langmuir–Freundlich models, the Hill cooperative binding model, and others to describe enzyme adsorption and binding to the substrate (Jalak and Väljamäe 2014). In our study, a reasonable fit with R^2^ values of 0.9–0.96 was obtained using the standard Langmuir binding model.

### SPR measurements to study cellulase binding to cellulose and lignin films

First, a method for the regeneration of the SPR probe film surface had to be developed to remove bound protein of previous measurements from the lignin or cellulose films. Different approaches including high ionic strength (5 M NaCl or 4 M MgCl), low pH (10 mM glycine HCl, pH 2.0), high pH (10 mM NaOH), and the use of a detergent (0.5% SDS) were tested. The test was performed using a set of different cellulolytic enzymes as well as enzymes involved in lignin degradation. Therefore, CBHI, glyoxal oxidase, pyranose 2-oxidase, laccase and cellobiose dehydrogenase were used for initial testing procedures. The best results were obtained by using a two-step procedure with 5 M NaCl followed by 10 mM glycine HCl pH 2.0 with a contact time of 30 s, which was then employed to regenerate the SPR probe after each measurement.

The thickness of the cellulose and lignin films was determined by AFM by carefully removing part of the film with a razor blade and measuring the height difference between the modified and unmodified surface (Fig. S4). Furthermore, the thickness of the cellulose or lignin films was calculated from the SPR data from the average of the signal from four flow channels according to Eqs. 1 and2 (Table 3). A calibration curve was determined using different concentrations of glycerol with known refractive indices (Fig. S5).

**Table 3:** Evaluation of film thickness from SPR measurements and AFM imaging. The thicknesses were calculated according to Eq. 2 estimating the densities and refractive indices of lignin and cellulose the as reported previously. The refractive index of the buffer solvent was calculated to be 1.334

	SPR					AFM
	Response units[RU]	Refractive index	Density[g cm^−3^]	Adsorbed mass[mg m^−2^]	Film thickness [nm]	Film thickness [nm]
OSL	22690 ± 1320	1.61^(1)^	1.30^(3)^	22.6 ± 1.3	17.4 ± 1.0	23.2 ± 4.0
MWL	17660 ± 1170	1.61^(1)^	1.30^(3)^	15.4 ± 1.0	11.8 ± 0.8	14.6 ± 2.4
Cellulose	24890 ± 2670	1.47^(2)^	1.60^(4)^	58.2 ± 6.2	36.4 ± 3.9	24.9 ± 6.1

^(1)^(Norgren et al. 2006)

^(2)^(Kasarova et al. 2007)

^(3)^(Triwulandari et al. 2019)

^(4)^(Daicho et al. 2020)

The thickness of the lignin-modified surfaces found by AFM were 23.2 nm and 14.6 nm for OSL and MWL, respectively, and are higher than the values obtained from SPR measurements. This could possibly be explained by the removal of not tightly bound lignin residues from the SPR probe film surface during the initial equilibration in running buffer and regeneration. The OSL films were slightly thicker than the MWL films. Film thickness as well as roughness potentially depend on the size distribution of lignin fragments, their composition, and their degree of branching. Pereira et al. observed that films prepared from spruce or wheat straw lignin appeared rougher and from spruce also thicker than films from eucalyptus wood, putatively because of the higher amount of syringyl-residues in eucalyptus lignin. Therefore, more linear structures and less branching is found in this type of lignin influencing the resulting films to be smoother (Pereira et al. 2017). In our study, the differences in thickness and roughness between the two types of lignin can also be explained by the feedstock and different extraction and pretreatment procedures. Cellulose showed a thickness of 24.9 nm when measured by AFM while a value of 36.4 nm was calculated from the SPR response. The values of the dry cellulose films determined by AFM are in good agreement with previous studies reporting 28 nm and 30 nm thick cellulose films in the dry state using a similar procedure (Eriksson et al. 2005a, 2005b).

The higher value obtained by SPR data could reflect the swelling of the cellulose film when exposed to the aqueous buffer. However, a precise quantification of the degree of swelling is not feasible by comparing data obtained by AFM and SPR measurements because of the limited comparability of the values obtained using different methods for the determination of film thicknesses in the nano-scale (Sczech and Riegler 2006).

Swelling of cellulose films prepared by spin coating techniques using different solvents has been reported and was shown to be more pronounced for amorphous films produced using DMAc/LiCl. Using quartz crystal microbalance measurements Aulin et al. determined the mass of adsorbed water in the cellulose film prepared from DMAc/LiCl as 48% of the swollen film (Aulin et al. 2009). Another study reports even more pronounced swelling behavior deduced from the observed decrease in the refractive indices upon buffer exposition resulting in an increase in the film thickness by a factor of 2.5 (Eriksson et al. 2005a).

**Fig. 5:**
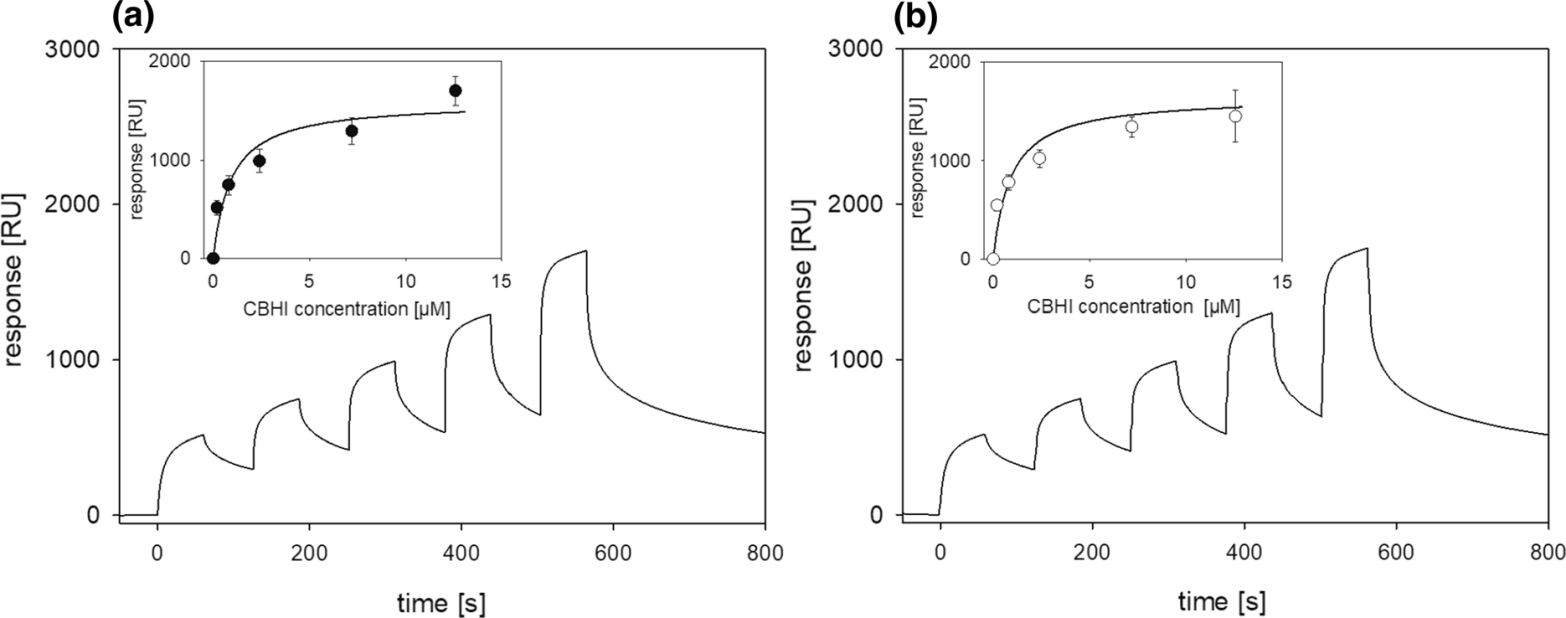
Binding of CBHI (**a**) and deglycosylated CBHI (**b**) to cellulose films was performed as single cycle kinetic (SCK) experiment with five injections of increasing enzyme concentration (0.2 µM, 0.8 µM, 2.4 µM, 7.2 µM and 12.6 µM). From the six performed measurements using two flow channels an averaged sensogram was calculated, the data in the inset are averaged from all six measurements

The regeneration of cellulose from DMAc/LiCl results in the formation of mainly amorphous cellulose and only a minor proportion of cellulose II (Aulin et al. 2009). Despite this difference to cellulose found naturally in the plant cell wall, cellulolytic enzymes within Celluclast as well as isolated cellobiohydrolases or endoglucanases have been shown to be capable of the degradation of artificial cellulose films (Eriksson et al. 2005a; Josefsson et al. 2008; Suchy et al. 2011). In particular, endoglucanases have been shown to degrade amorphous cellulose films efficiently and cause pronounced swelling of the model film as demonstrated by quartz crystal microbalance measurements (Suchy et al. 2011). To reduce the total contact time and to avoid fast removal of the film upon exposure to the enzymes, the binding studies with cellulose were performed as single cycle kinetic (SCK) measurements. This technique has been used to study binding of lytic polysaccharide monooxygenase (LPMO) to cellulosic surfaces and to investigate the role of the CBM (Laurent et al. 2019). No significant difference between the untreated and the deglycosylated CBHI was observed by SPR measurements showing that the removal of N-glycans does only slightly affect substrate binding (Fig. 5). Furthermore, no significant difference between the *K*_D_ values of different enzyme preparations was observed, which is in good agreement with the data obtained from the binding studies using PASC and MCC. The maximal surface loading was calculated as 1.8 mg m^−2^ for the untreated and 1.7 mg m^−2^ for the deglycosylated CBHI (Table 4).

**Table 4:** Apparent dissociation constants (*K*_D_) and maximal surface loading (Γ_max_) determined by SPR on cellulose. The density of the adsorbed protein layer and the refractive index were estimated as 1.32 g cm^−3^ and 1.57, respectively

Enzyme preparation	*K*_D_ [µM]	R_max_ [RU]	*Γ*_max_ [mg enzyme m^−2^]
Untreated CBHI	0.91 ± 0.17	1611 ± 68	1.82 ± 0.04
Deglycosylated CBHI	0.78 ± 0.15	1528 ± 70	1.72 ± 0.05

The surface properties and hydrophobicity of lignocellulolytic enzymes is crucial for their tendency to adsorption to lignin. Although some reports also highlight ionic interaction and hydrogen bonding that can contribute to binding to lignin, this undesired interaction is suggested to be caused mainly by hydrophobic interactions. CBHI has been shown to have a higher affinity to lignin than xylanases, EGs and β-glucosidases and especially the CBM is thought to contribute to this undesired non-productive binding to the lignin fraction of plant biomass (Berlin et al. 2006; Guo et al. 2014). Investigations based on NMR measurements using the isolated CBM of CBHI and artificial lignin oligomers verified the strong interactions between mainly of tyrosine residues of the CBM and the aromatic lignin by hydrophobic interactions as well as π-π stacking (Tokunaga et al. 2020).

A factor that modulates the solubility and hydrophobicity of enzymes is the posttranslational modification with glycosides. Within the amino acid sequence of CBHI from *T. reesei* four Asn-Xaa-(Ser/Thr) sequons–Asn45, Asn64, Asn270 and Asn384–could be potential sites of N-glycosylation. Applying capillary liquid chromatography coupled electrospray mass spectrometry (cLC-ESMS), Hui et al. found that only Asn270 harbours a high-mannose type glycan while Asn45 and Asn384 are only modified with single GlcNAc moieties. A large variability and heterogeneity in the N-glycosylation patterns of Cel7A is reported which is proposed to depend on the fungal strains and cultivation processes influencing the level of endoglucosidase H or endoglucosidase F activity in the culture fluid. Additionally, Ser and Thr residues found in the linker region between the catalytic core region and the CBM were shown to be heterogeneously O-glycosylated (Klarskov et al. 1997; Hui et al. 2001; Stals et al. 2004; Amore et al. 2017). Although some reports based on the results of site directed mutagenesis studies targeting the N-glycosylation sites of CBHI from *T. reesei* point out that N-glycosylation is important for its thermal stability (Qi et al. 2014; Amore et al. 2017) and the presence of large glycans at the catalytic domain interferes with cellulose binding and catalytic processivity (Jeoh et al. 2008; Adney et al. 2009), the results are not completely conclusive. Amore et al. further highlighted the importance of N-glycosylation as well as O-glycosylation within the linker region for protein stability against proteolytic degradation by the investigation of a large set of mutated CBHI variants (Amore et al. 2017). MD simulations of the interaction of CBHI with crystalline cellulose fibrils further revealed the significance of O-glycosylation for efficient substrate binding (Payne et al. 2013; Amore et al. 2017). Using different expression host as well as N-glycan knockout variants, Kołaczkowski et al. demonstrated that large N-linked glycans do interfere with binding to cellulose. The strongest effect was reported for crystalline cellulose (Avicel) and resulted in a more then twofold increase of the surface loading upon complete N-glycan removal taking into account the density of potential attack sites (Kołaczkowski et al. 2020). On regenerated amorphous cellulose, this effect was much less pronounced which explains the similar substrate affinity we found by SPR measurements using non-crystalline cellulose films.

**Fig. 6:**
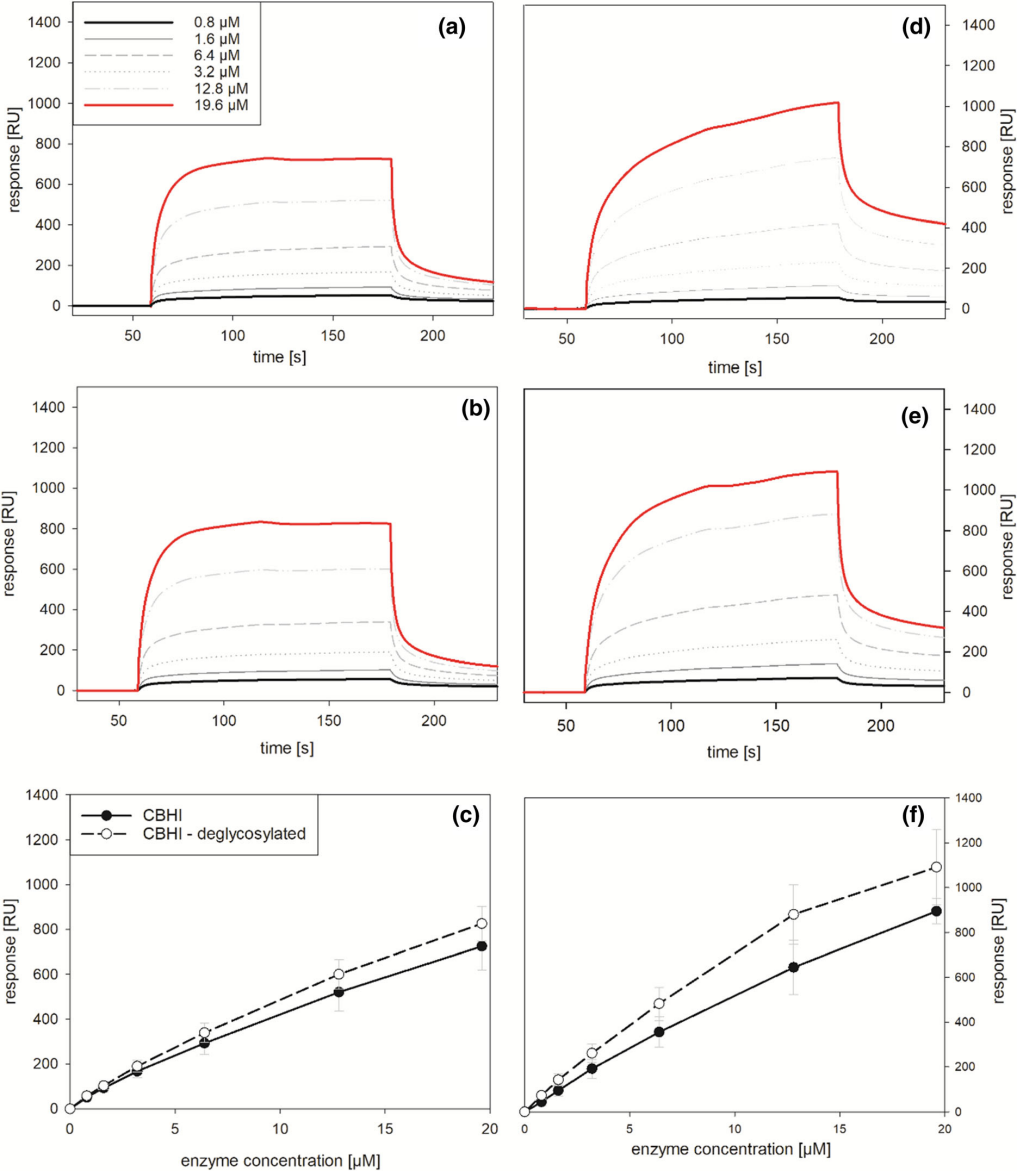
Binding of CBHI to lignin. Sensograms of multicycle kinetic (MCK) measurements with CBHI (**a**, **d**) and deglycosylated CBHI (**b**, **e**) were measured with model films prepared from OSL (**a**, **b**, **c**) and MWL (**d**, **e**, **f**). The response after 120 s of contact time vs. enzyme concentration (**c**, **f**) is presented as average of four measurements using two independent flow channels

In our study, the removal of N-linked glycans results in a slightly stronger binding of deglycosylated CBHI to lignin model films from OSL as well as MWL than the untreated enzyme (Fig. 6). The only small difference can be partly explained by the low degree of glycosylation of CBHI and additionally by the fact that the core GlcNAc unit is not removed by the treatment with Endo Hf. Analysis by SDS-PAGE showed a decrease in molecular weight from 60.6 kDa to 59.0 kDa upon deglycosylation with Endo Hf (Fig. S2).

A slightly higher surface loading could be observed with lignin films prepared from MWL and also the binding was less reversible compared with the films prepared from OSL. MWL is produced by a milder form of extraction procedure yielding a lignin preparation that is more representative for the natural form found in the plant cell wall. Those results demonstrate that the extraction process influences the adsorption properties of lignin. However, the higher adsorption to MWL films could also be explained by the difference between lignin from beech and spruce wood. Previous studies concluded that the S/G ratio influences the adsorption of enzymes and therefore the lower saccharification yield with G-rich lignin showing a higher adsorption capacity (Guo et al. 2014). However, the adsorption capacity and maximal surface loading is also influenced by the surface roughness which was found to be higher for MWL films compared to OSL films. The ratio between reversible and irreversible binding under the conditions used was higher for OSL compared to MWL. At the highest enzyme concentration used in this setup (19.6 µM CBHI) a maximal surface loading of 0.8 mg m^−2^ on OSL versus 1.2 mg m^−2^ on MWL films was observed with CBHI.

**Fig. 7:**
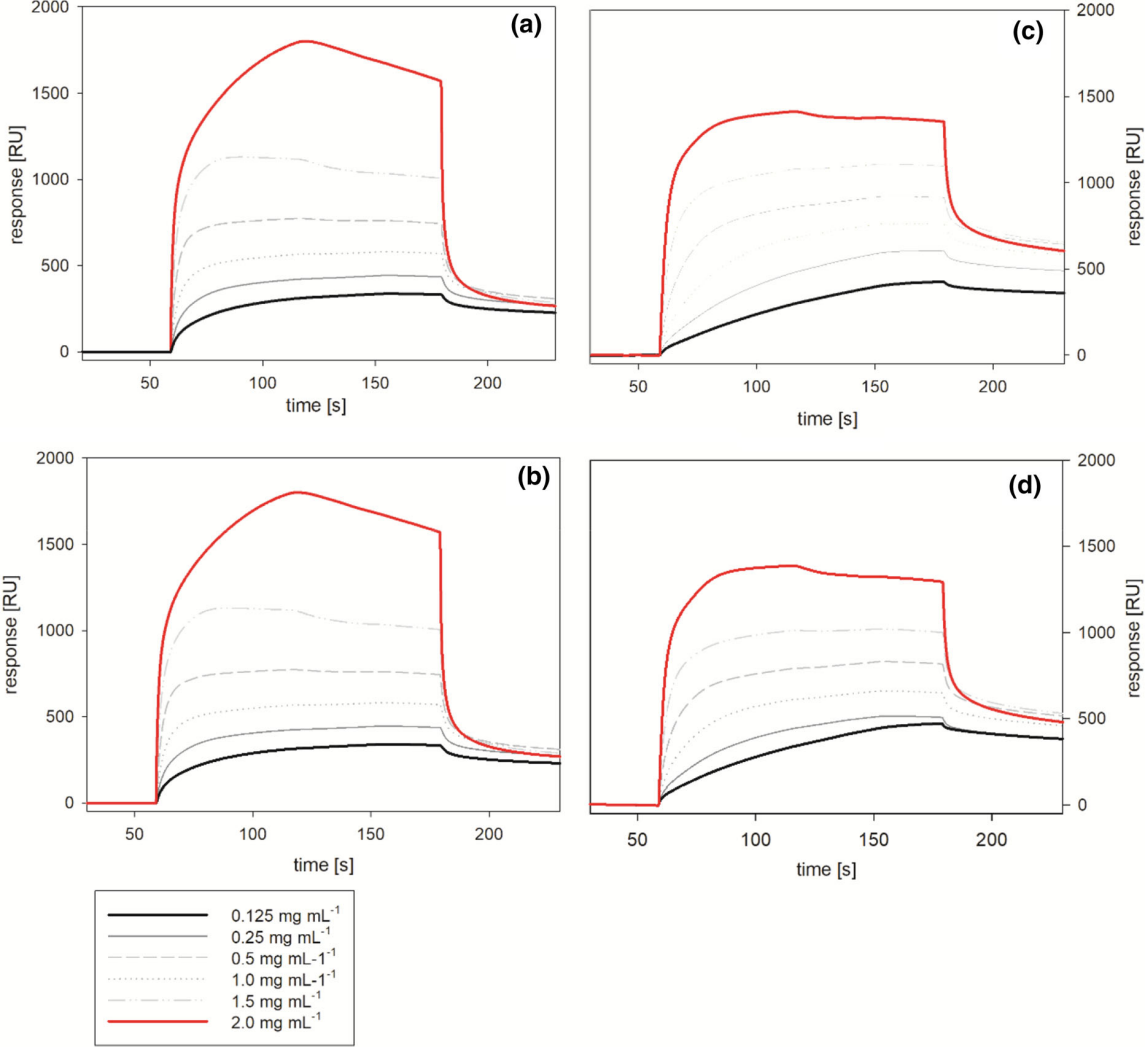
Binding of Celluclast to lignin. The sensograms of multicycle kinetic (MCK) measurements with Celluclast (**a**, **c**) and of deglycosylated Celluclast enzymes (**b**, **d**) were measured with model films prepared from OSL (**a**, **b**) and MWL (**c**, **d**)

The same method was applied to study binding of the multienzyme system Celluclast (Fig. 7). The results confirmed the observation made with the isolated CBHI and no significant effect upon removal of N-glycosylation could be identified. This can be explained with the lower number of potential glycosylation sites found in the sequences of the other main components of Celluclast: CBHII and EGs I–V. Therefore, deglycosylation has a higher impact on the isolated CBHI enzyme. Particularly with OSL, different phases in the adsorption process could be observed as a result of the different affinities of the individual enzymes. At the highest concentration used (2 mg mL^−1^) the maximum surface loading was reached approximately 60 s after the injection of the enzyme preparations followed by a slow decrease in the signal. Especially with the MWL modified sensor tested with lower enzyme concentrations (0.125–0.25 mg mL^−1^) a very slow binding to the substrate was found and the equilibrium could not be reached within 120 s of contact time. This slow adsorption is typical for unspecific binding processes with a low specific affinity. At a 2 mg mL^−1^ enzyme concentration, the surface loading calculated from the maximal observed response was 2.1 and 1.6 mg m^−2^ of adsorbed enzyme was determined for OSL and MWL. Using a different buffer system with a higher ionic strength of 50 mM, a surface loading of 2.2 mg m^−2^ on MWL films from spruce using the CTec2 has been reported which is in good agreement with our findings (Pereira et al. 2017). Particularly with OSL, different phases within the adsorption process could be observed as a result of the different affinities of the individual components of the enzyme mixture. Interestingly, we observed a higher surface loading on OSL compared to MWL using Celluclast. This result indicates that the lignin composition influences the adsorption capacity as well as the fact that different enzymes show different binding tendency for certain types of lignin.

## Conclusion

The characterization of enzyme binding constants by enzyme addition to a substrate dispersion consumes a lot of time and effort. Although films produced from cellulose or lignin are a simplified model for the representation of natural biomass, artificial films are valuable tools to investigate the heterogeneous catalysis of lignocellulolytic enzymes, their binding affinities as well as their tendency for unspecific adsorption to lignin. The model films used in this study were shown to be suitable for the measurement of binding behaviour by SPR. The dissociation constants (*K*_D_) estimated by SPR of ~ 1 µM for CBHI onto cellulose are in good agreement with the values determined by UV–Vis spectrometry based measurements and previously reported values. Furthermore, we were able to compare the binding behaviour of Celluclast and isolated CBHI before and after removal of N-glycosylation. Only a minor increase in the binding affinity and surface loading could be detected upon deglycosylation, while the activity and the binding to cellulose was not affected significantly. Based on those results we conclude that the main role of N-linked glycosylation is not prevailingly to prevent non-productive binding to lignin nor is it changing productive binding to cellulosic substrates significantly. Only moderate differences in non-productive binding to MWL or OSL could be identified which showcases the relevance of this mechanism in the enzymatic processing of native as well as pretreated lignocellulosic feedstocks.

SPR based methods are routinely applied for the high throughput screening of receptor ligands, antibodies and other pharmaceutically relevant compounds. We believe that an SPR method utilizing model films prepared from cellulose, hemicellulose or lignin will reduce analysis time and thereby aid the screening and characterization of enzymes for biopolymer depolymerization.

## Supplementary Information

Below is the link to the electronic supplementary material.

Supplementary file1 (PDF 1085 KB)
