# Supplementary material for: Non-productive binding of cellobiohydrolase i investigated by surface plasmon resonance spectroscopy
Source: Cellulose (Lond). 2021 Aug 25;28(15):9525–45. doi: 10.1007/s10570-021-04002-6 (PMC8550311; doi:10.1007/s10570-021-04002-6)
Supplement: Supplementary file 1 — Supplementary file1 (PDF 1085 KB) [file 10570_2021_4002_MOESM1_ESM.pdf]

Non-Productive Binding of Cellobiohydrolase I Investigated by Surface Plasmon Resonance Spectroscopy

Supplemental Information

Florian Csarman[1], Claudia Gusenbauer[2], Lena Wohlschlager[1], Gijs van Erven[3], Mirjam A. Kabel[3], Johannes Konnerth[2], Antje Potthast[4], Roland Ludwig[1]*

[1] Department of Food Science and Technology, Biocatalysis and Biosensing Laboratory, BOKU University of Natural Resources and Life Sciences, Muthgasse 18, 1190 Vienna, Austria

[2] Department of Materials Sciences and Process Engineering, Institute of Wood Technology and Renewable Materials, BOKU - University of Natural Resources and Life Sciences, Konrad-Lorenz-Straße 24, 3430, Tulln, Austria

[3] Laboratory of Food Chemistry, Wageningen University & Research, Bornse Weilanden 9, 6708 WG Wageningen, The Netherlands

[4] Department of Chemistry, Division of Chemistry of Renewable Resources, BOKU - University of Natural Resources and Life Sciences, Konrad-Lorenz-Straße 24, A-3430 Tulln, Austria

Florian Csarman: florian.csarman@boku.at

Claudia Gusenbauer: claudia.gusenbauer@boku.ac.at

Lena Wohlschlager: lena.wohlschlager@boku.ac.at

Mirjam A. Kabel: mirjam.kabel@wur.nl

Antje Potthast: antje.potthast@boku.ac.at

*Roland Ludwig: roland.ludwig@boku.ac.at

*corresponding author

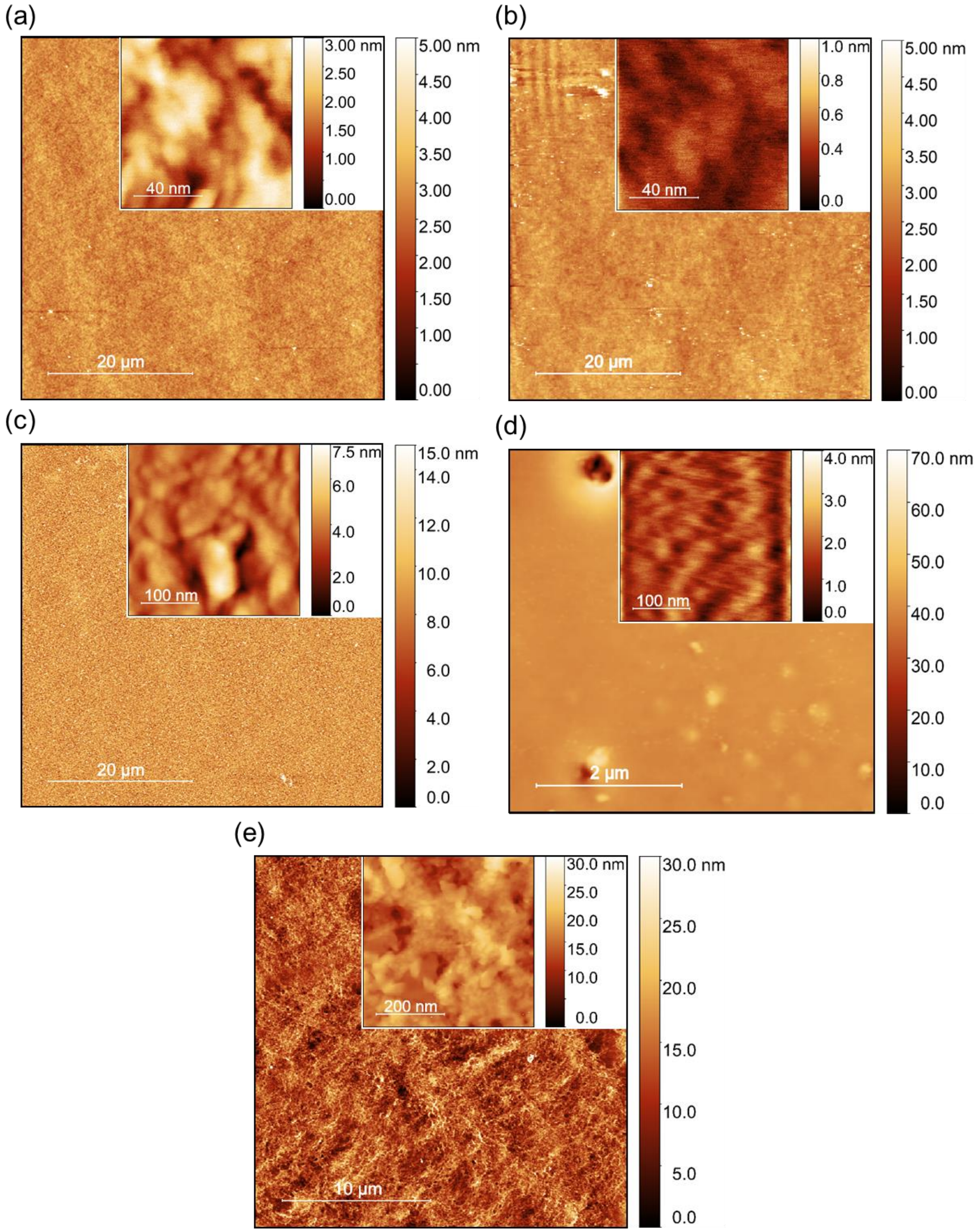

Fig. S1: AFM images of piranha solution treated gold surface (a) and glass surface (b), PDADMAC (c) or polysterene (d) coated gold surface and cellulose modified gold surface (e).

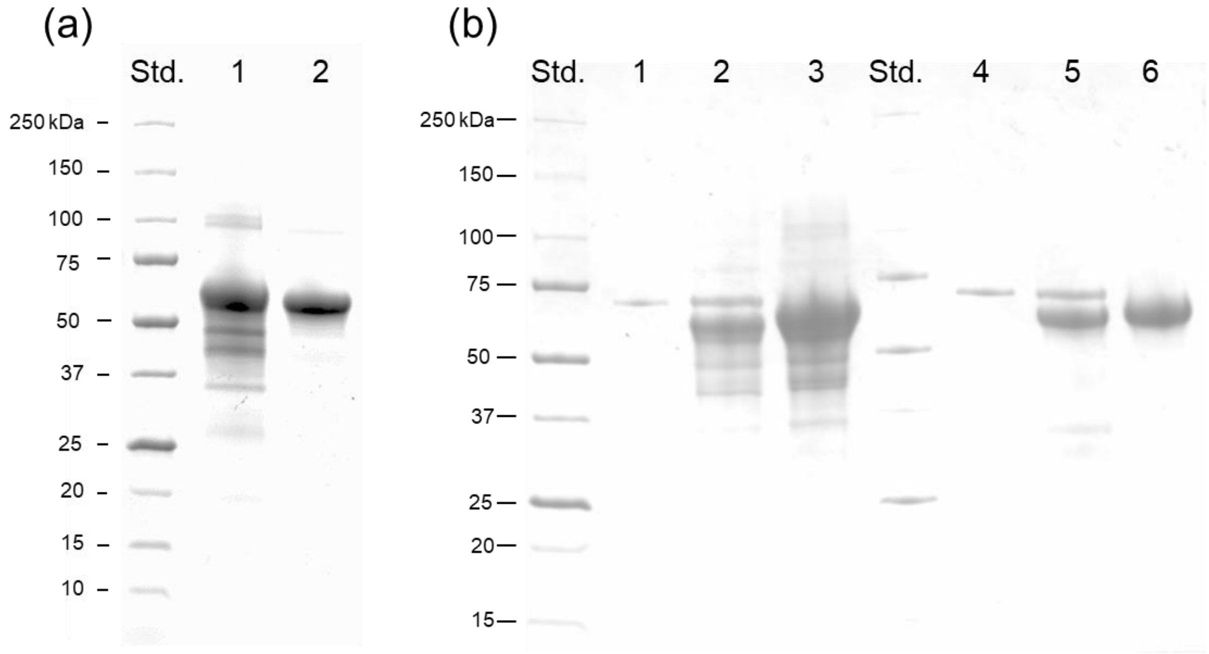

Fig. S2: SDS-PAGE of the purification of CBHI (a) showing Celluclast (lane 1) and purified CBHI (lane 2) and of the deglycosylation using Endo Hf (b) showing pure Endo-Hf (lane 1 and 4), deglycosylated Celluclast (lane 2) untreated Celluclast (lane 3) deglycosylated CBHI (lane 5) and untreated CBHI (lane 6).

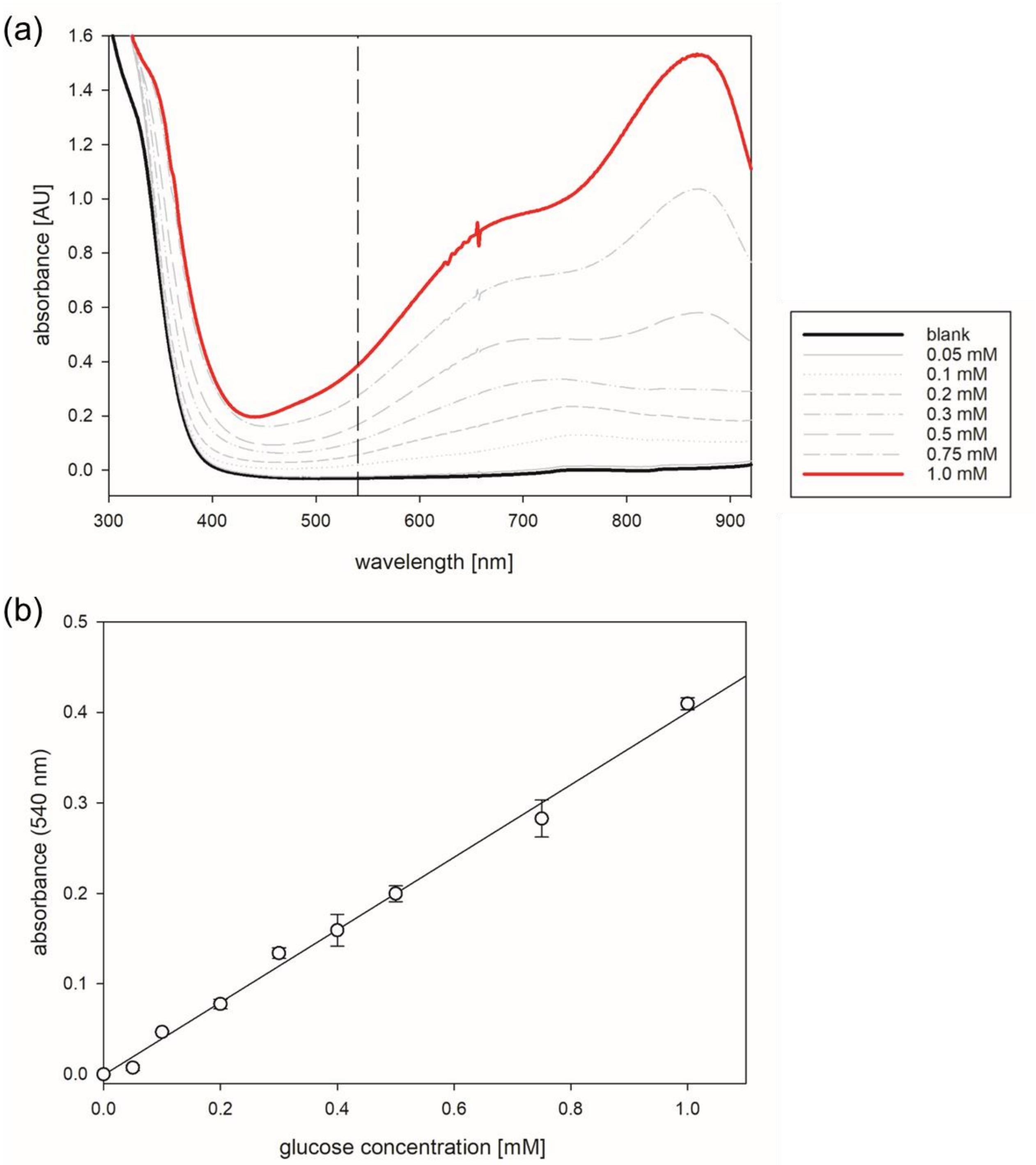

Fig. S3: Calibration curve for the determination of reducing sugars using the Nelson-Somogyi assay. Glucose standards from 0.05 mM to 1.0 mM were used and spectra were recorded from 300 to 900 nm (a). The absorbance at 540 nm was used for quantitation (b). Measurements were performed in triplicates.

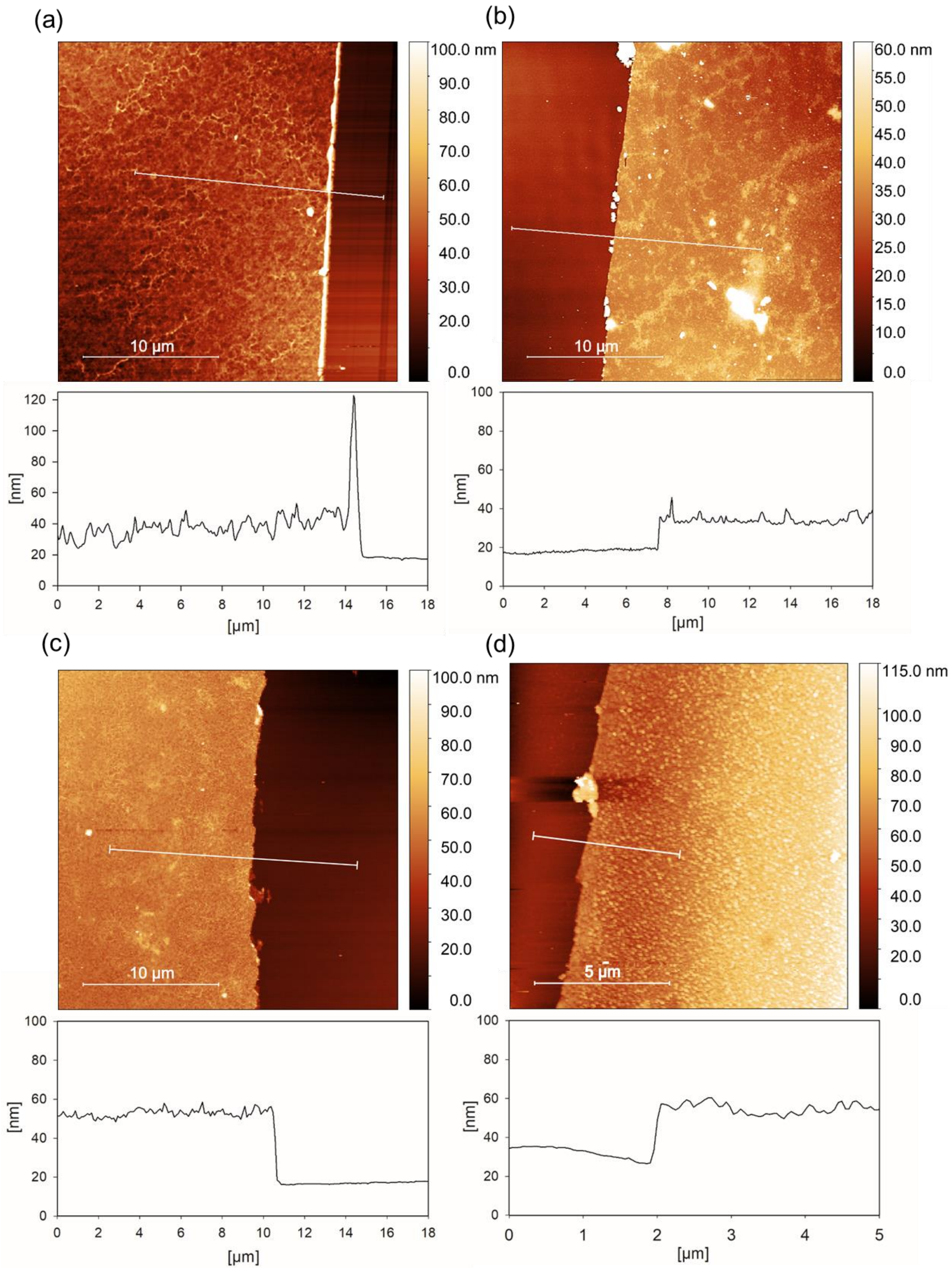

Fig. S4: Determination of height-profiles by AFM for the evaluation of the thickness of cellulose films on gold (a), cellulose films on glass (c) and lignin films on gold prepared from MWL (b) and OSL (d).

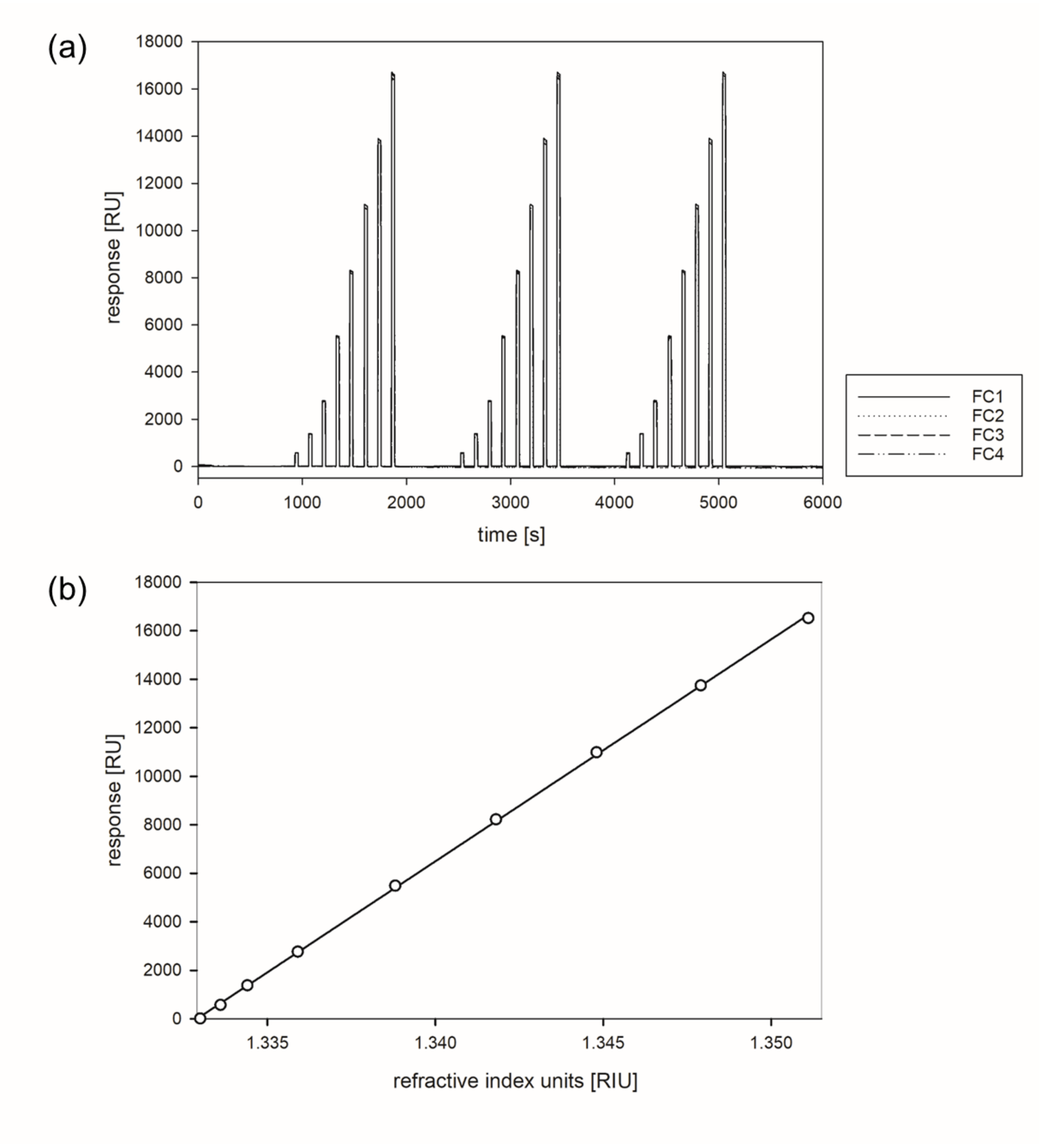

Fig. S5: Determination of sensitivity factor for the quantification of adsorbed mass to the sensor surface. Experiments were performed on four flow channels (a). Glycerol solutions (0, 0.5, 1.25, 2.5, 5.0, 7.5, 10, 12.5, and 15 % wt.) with known refractive index (η) for the determination of the standard curve (b). All standard errors were below 1%.

Table SI 1: Preparation procedures and characterization of cellulose and lignin model films prepared by spin coating techniques.

	Cellulose source	Method of deposition	Substrate	Film thickness [nm]	RMS roughness [nm]	Contact angle [deg]		Surface free energy [mN m^{-1}]			Reference
						Water	CH_2I_2	Total	Polar	Disperse	
Cellulose	Avicel	dissolution in DMAc/LiCl	silica	28	4.8	33	n.d.	n.d.	n.d.	n.d.	(Eriksson et al. 2005)
	cellulose xanthate	regeneration from cellulose xanthate	gold	20 - 407	4.5 - 5.6	30 - 37	32 - 34	66.8	42.5	23.1	(Weißl et al. 2018)
	trimethylsilylcellulose	regeneration from TMSC	silica	24	0.78	51.5	n.d.	60.2	n.d.	n.d.	(Mohan et al. 2011)
	trimethylsilylcellulose	regeneration from TMSC	gold	19.5	3						(Kontturi et al. 2003)
	Avicel	dissolution in DMAc/LiCl	silica	0.5 - 10	< 2	25-35	n.d.	n.d.	n.d.	n.d.	(Sczech and Riegler 2006)
	MCC (Merck)	MCC dissolved in DMAc/LiCl	gold	24.9	5.6	30.5	45.1	67.1	30.4	36.7	this work
	MCC (Merck)	MCC dissolved in DMAc/LiCl	glass	29.8	5.4	30	43.3	67.1	39.0	27.8	this work

	Lignin source	Method of deposition	Substrate	Film thickness [nm]	RMS roughness [nm]	Contact angle [deg]		Surface free energy [mN m^{-1}]			Reference
						Water	CH_2I_2	Total	Polar	Disperse	
Lignin	MWL from eucalyptus	dissolution in dioxane/water	gold	9	4.15	66	n.d.	n.d.	n.d.	n.d.	(Pereira et al. 2017)
	MWL from wheat straw	dissolution in dioxane/water	gold	9	2.14	69	n.d.	n.d.	n.d.	n.d.	(Pereira et al. 2017)
	Kraft lignin fom softwood	dissolution in 1 M NH_4OH	silica	6.9	1.2	54	n.d.	n.d.	n.d.	n.d.	(Pereira et al. 2017)
	OSL from beech wood	dissolution in 1 M NH_4OH	silica	n.d.	1.5	58	n.d.	n.d.	n.d.	n.d.	(Borrega et al. 2020)
	soda lignin from wheat straw	dissolution in 1 M NH_4OH	silica	11.3	1.6	43	n.d.	n.d.	n.d.	n.d.	(Borrega et al. 2020)
	soda oxidized lignin from wheat straw	dissolution in 1 M NH_4OH	silica	9.5	0.5	n.d.	n.d.	n.d.	n.d.	n.d.	(Borrega et al. 2020)
	lignosulfonate from softwood	dissolution in 1 M NH_4OH	silica	5.3	0.9	n.d.	n.d.	n.d.	n.d.	n.d.	(Borrega et al. 2020)
	Ecohelix	dissolution in 1 M NH_4OH	silica	6.3	0.5	n.d.	n.d.	n.d.	n.d.	n.d.	(Borrega et al. 2020)
	Kraft lignin from spruce	dissolution in 1 M NH_4OH	silica	50-60	0.93	46	51	57.1	23.4	33.7	(Notley and Norgren 2010)
	MWL pine	dissolution in acetone/ water (9:1)	silica	30-60	1.31	52.5	23	58.8	14.3	44.5	(Notley and Norgren 2010)
	MWL eucalyptus	dissolution in acetone/ water (9:1)	silica	30-60	1.38	55.5	27	57	13.1	43.9	(Notley and Norgren 2010)
	Kraft lignin from softwood	dissolution in dioxane/water (9:1)	silica	23.1 – 104.6	1.01 – 1.39	46	n.d.	n.d.	n.d.	n.d.	(Norgren et al. 2006)
	OSL from beech wood	dissolution in dioxane/water	gold	23.2	4.4	67.6	34.2	49.9	7.5	42.4	this work
	MWL from spruce	dissolution in dioxane/water	gold	14.6	8.3	68.3	41.6	45.0	6.2	38.8	this work
	OSL from beech wood	dissolution in dioxane/water	glass	n.d.	n.d.	64	39.6	49.9	10	40	this work
	MWL from spruce	dissolution in dioxane/water	glass	n.d.	n.d.	62.2	37.2	49.5	8.5	41	this work

References

Borrega M, Päärnilä S, Greca LG, Jääskeläinen AS, Ohra-Aho T, Rojas OJ, Tamminen T (2020) Morphological and wettability properties of thin coating films produced from technical lignins. Langmuir 36:9675–9684 . https://doi.org/10.1021/acs.langmuir.0c00826

Eriksson J, Malmsten M, Tiberg F, Hønger T (2005) Enzymatic degradation of model cellulose films. 284:99–106 . https://doi.org/10.1016/j.jcis.2004.10.041

Kontturi E, Thüne PC, Niemantsverdriet JW (2003) Novel method for preparing cellulose model surfaces by spin coating. Polymer (Guildf) 44:3621–3625 . https://doi.org/10.1016/S0032-3861(03)00283-0

Mohan T, Kargl R, Doliška A, Vesel A, Köstler S, Ribitsch V, Stana-Kleinschek K (2011) Wettability and surface composition of partly and fully regenerated cellulose thin films from trimethylsilyl cellulose. J Colloid Interface Sci 358:604–610 . https://doi.org/10.1016/j.jcis.2011.03.022

Norgren M, Notley SM, Majtnerova A, Gellerstedt G (2006) Smooth model surfaces from lignin derivatives. I. Preparation and characterization. Langmuir 22:1209–1214 . https://doi.org/10.1021/la052284c

Notley SM, Norgren M (2010) Surface energy and wettability of spin-coated thin films of lignin isolated from wood. Langmuir 26:5484–5490 . https://doi.org/10.1021/la1003337

Pereira A, Hoeger IC, Ferrer A, Rencoret J, Del Rio JC, Kruus K, Rahikainen J, Kellock M, Gutiérrez A, Rojas OJ, Rio JC, Kruus K, Rahikainen J, Kellock M, Gutie A, Rojas OJ, Del Rio JC, Kruus K, Rahikainen J, Kellock M, Gutiérrez A, Rojas OJ (2017) Lignin Films from Spruce, Eucalyptus, and Wheat Straw Studied with Electroacoustic and Optical Sensors: Effect of Composition and Electrostatic Screening on Enzyme Binding. Biomacromolecules 18:1322–1332 . https://doi.org/10.1021/acs.biomac.7b00071

Sczech R, Riegler H (2006) Molecularly smooth cellulose surfaces for adhesion studies. J Colloid Interface Sci 301:376–385 . https://doi.org/10.1016/j.jcis.2006.05.021

Weißl M, Niegelhell K, Reishofer D, Zankel A, Innerlohinger J, Spirk S (2018) Homogeneous cellulose thin films by regeneration of cellulose xanthate: properties and characterization. Cellulose 25:711–721 . https://doi.org/10.1007/s10570-017-1576-3
